# *N*′-(3-Bromo-5-chloro-2-hydroxy­benzyl­idine)-2-hydroxy­benzohydrazide

**DOI:** 10.1107/S1600536808038634

**Published:** 2008-11-26

**Authors:** Wagee A. Yehye, Azhar Ariffin, Noorsaadah A. Rahman, Seik Weng Ng

**Affiliations:** a Department of Chemistry, University of Malaya, 50603 Kuala Lumpur, Malaysia

## Abstract

In the approximately planar title mol­ecule, C_14_H_10_BrClN_3_O_2_, the dihedral angle between the aromatic ring planes is 5.79 (12)°. The conformation is stabilized by intra­molecular O—H⋯N and N—H⋯O hydrogen bonds and an inter­molecular O—H⋯O link leads to chains in the crystal propagating in [001].

## Related literature

For similar Schiff bases, see: Hu *et al.* (2005 ▶); Wu *et al.* (2006 ▶); Yehye *et al.* (2008*a* ▶,*b* ▶).
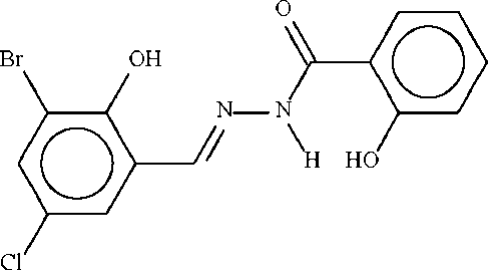


## Experimental

### 

#### Crystal data

C_14_H_10_BrClN_2_O_3_
*M* *_r_* = 369.60
Monoclinic, 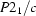

*a* = 15.8387 (3) Å
*b* = 6.9319 (1) Å
*c* = 12.9951 (3) Å
β = 106.461 (1)°
*V* = 1368.28 (5) Å^3^
*Z* = 4
Mo *K*α radiation
μ = 3.21 mm^−1^
*T* = 100 (2) K
0.30 × 0.20 × 0.05 mm

#### Data collection

Bruker SMART APEX CCD diffractometer
Absorption correction: multi-scan (*SADABS*; Sheldrick, 1996 ▶) *T*_min_ = 0.446, *T*_max_ = 0.856
12350 measured reflections
3136 independent reflections
2545 reflections with *I* > 2σ(*I*)
*R*_int_ = 0.038

#### Refinement

*R*[*F* ^2^ > 2σ(*F* ^2^)] = 0.029
*wR*(*F* ^2^) = 0.077
*S* = 1.02
3136 reflections
202 parameters
3 restraints
H atoms treated by a mixture of independent and constrained refinement
Δρ_max_ = 0.43 e Å^−3^
Δρ_min_ = −0.41 e Å^−3^

### 

Data collection: *APEX2* (Bruker, 2007 ▶); cell refinement: *SAINT* (Bruker, 2007 ▶); data reduction: *SAINT*; program(s) used to solve structure: *SHELXS97* (Sheldrick, 2008 ▶); program(s) used to refine structure: *SHELXL97* (Sheldrick, 2008 ▶); molecular graphics: *X-SEED* (Barbour, 2001 ▶); software used to prepare material for publication: *publCIF* (Westrip, 2008 ▶).

## Supplementary Material

Crystal structure: contains datablocks global, I. DOI: 10.1107/S1600536808038634/hb2857sup1.cif

Structure factors: contains datablocks I. DOI: 10.1107/S1600536808038634/hb2857Isup2.hkl

Additional supplementary materials:  crystallographic information; 3D view; checkCIF report

## Figures and Tables

**Table 1:** Hydrogen-bond geometry (Å, °)

*D*—H⋯*A*	*D*—H	H⋯*A*	*D*⋯*A*	*D*—H⋯*A*
O1—H1*o*⋯O2^i^	0.83 (1)	1.76 (1)	2.591 (2)	175 (3)
O3—H3*o*⋯N2	0.84 (1)	1.88 (2)	2.632 (3)	149 (3)
N1—H1*n*⋯O1	0.87 (1)	1.90 (2)	2.614 (3)	139 (2)

Symmetry code: (i) 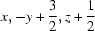.

**Fig. 1.**
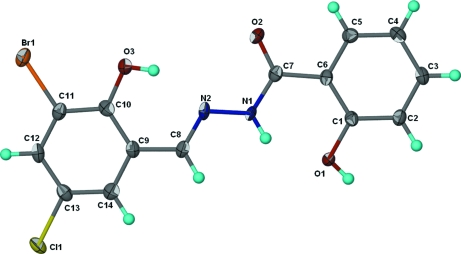
The molecular structure of (I) with atoms shown at the 70% probability level. Hydrogen atoms are drawn as spheres of arbitrary radiius.

## supplementary crystallographic information

### Comment

In the approximately planar title molecule, (I), (Fig. 1) the dihedral angle between the aromatic ring planes is 5.79 (12)°.   The conformation is stabilised by intramolecular O—H···N and N—H···O hydrogen bonds and an intermolecular O—H···O link leads to chains in the crystal (Table 1).

### Experimental

2-Hydroxybenzohydrazide(0.60 g, 4 mmol) and 3-bromo-5-chloro-2-ydroxybenzaldehyde (0.94 g, 4 mmol) were heated in ethanol (30 ml) for 2 h. The solvent was removed by evaporation and the resulting solid was recrystallized from ethanol to yield yellow plates of (I).

### Refinement

The carbon-bound H-atoms were placed in calculated positions (C–H = 0.95 Å) and refined as riding with *U*(H) = 1.2*U*(C). The oxygen- and nitrogen-bound H-atoms were located in a difference map, and were refined with distance restraints of O–H 0.84±0.01 and N–H 0.88±0.01 Å. Their U_iso_ values were freely refined.

### Crystal data

**Table:**

C_14_H_10_BrClN_2_O_3_	*F*_000_ = 736
*M**_r_* = 369.60	*D*_x_ = 1.794 Mg m^−^^3^
Monoclinic, *P*2_1_/*c*	Mo *K*α radiation λ = 0.71073 Å
Hall symbol: -P 2ybc	Cell parameters from 3691 reflections
*a* = 15.8387 (3) Å	θ = 3.2–28.2º
*b* = 6.9319 (1) Å	µ = 3.21 mm^−^^1^
*c* = 12.9951 (3) Å	*T* = 100 (2) K
β = 106.461 (1)º	Plate, yellow
*V* = 1368.28 (5) Å^3^	0.30 × 0.20 × 0.05 mm
*Z* = 4	

### Data collection

**Table:**

Bruker SMART APEX CCD diffractometer	3136 independent reflections
Radiation source: fine-focus sealed tube	2545 reflections with *I* > 2σ(*I*)
Monochromator: graphite	*R*_int_ = 0.038
*T* = 100(2) K	θ_max_ = 27.5º
ω scans	θ_min_ = 1.3º
Absorption correction: Multi-scan(SADABS; Sheldrick, 1996)	*h* = −20→20
*T*_min_ = 0.446, *T*_max_ = 0.856	*k* = −9→8
12350 measured reflections	*l* = −16→16

### Refinement

**Table:**

Refinement on *F*^2^	Secondary atom site location: difference Fourier map
Least-squares matrix: full	Hydrogen site location: difmap and geom
*R*[*F*^2^ > 2σ(*F*^2^)] = 0.029	H atoms treated by a mixture of independent and constrained refinement
*wR*(*F*^2^) = 0.077	*w* = 1/[σ^2^(*F*_o_^2^) + (0.0353*P*)^2^ + 1.3959*P*] where *P* = (*F*_o_^2^ + 2*F*_c_^2^)/3
*S* = 1.02	(Δ/σ)_max_ = 0.001
3136 reflections	Δρ_max_ = 0.43 e Å^−^^3^
202 parameters	Δρ_min_ = −0.41 e Å^−^^3^
3 restraints	Extinction correction: none
Primary atom site location: structure-invariant direct methods	

### Special details

**Table:**

Geometry. All e.s.d.'s (except the e.s.d. in the dihedral angle between two l.s. planes) are estimated using the full covariance matrix. The cell e.s.d.'s are taken into account individually in the estimation of e.s.d.'s in distances, angles and torsion angles; correlations between e.s.d.'s in cell parameters are only used when they are defined by crystal symmetry. An approximate (isotropic) treatment of cell e.s.d.'s is used for estimating e.s.d.'s involving l.s. planes.

### Fractional atomic coordinates and isotropic or equivalent isotropic displacement parameters (Å^2^)

**Table:**

	*x*	*y*	*z*	*U*_iso_*/*U*_eq_	
Br1	0.145097 (18)	0.43521 (4)	0.04463 (2)	0.02196 (9)	
Cl1	−0.04591 (4)	0.45367 (9)	0.33930 (5)	0.01932 (14)	
O1	0.48671 (11)	0.8436 (3)	0.72174 (14)	0.0186 (4)	
H1O	0.495 (2)	0.837 (5)	0.7879 (9)	0.033 (9)*	
O2	0.52114 (12)	0.6952 (3)	0.42779 (14)	0.0224 (4)	
O3	0.28679 (12)	0.5844 (3)	0.23371 (14)	0.0184 (4)	
H3O	0.3254 (15)	0.616 (4)	0.2897 (15)	0.027 (9)*	
N1	0.42237 (13)	0.7471 (3)	0.52022 (16)	0.0142 (4)	
H1N	0.4157 (17)	0.778 (4)	0.5825 (12)	0.015 (7)*	
N2	0.35545 (13)	0.6804 (3)	0.43620 (16)	0.0154 (4)	
C1	0.56768 (15)	0.8577 (4)	0.70555 (19)	0.0140 (5)	
C2	0.64075 (16)	0.9151 (4)	0.7881 (2)	0.0163 (5)	
H2	0.6343	0.9445	0.8569	0.020*	
C3	0.72242 (16)	0.9295 (4)	0.7702 (2)	0.0171 (5)	
H3	0.7720	0.9670	0.8271	0.021*	
C4	0.73279 (16)	0.8897 (4)	0.6701 (2)	0.0174 (5)	
H4	0.7890	0.9010	0.6581	0.021*	
C5	0.66035 (16)	0.8333 (4)	0.5875 (2)	0.0158 (5)	
H5	0.6674	0.8061	0.5187	0.019*	
C6	0.57684 (15)	0.8157 (3)	0.60395 (19)	0.0131 (5)	
C7	0.50521 (16)	0.7480 (3)	0.51062 (19)	0.0149 (5)	
C8	0.27901 (16)	0.6691 (4)	0.4507 (2)	0.0154 (5)	
H8	0.2706	0.7070	0.5174	0.018*	
C9	0.20470 (16)	0.5978 (3)	0.3648 (2)	0.0155 (5)	
C10	0.21190 (16)	0.5580 (3)	0.2611 (2)	0.0153 (5)	
C11	0.13777 (17)	0.4882 (4)	0.1845 (2)	0.0162 (5)	
C12	0.05895 (16)	0.4549 (3)	0.2073 (2)	0.0169 (5)	
H12	0.0095	0.4062	0.1538	0.020*	
C13	0.05363 (16)	0.4941 (4)	0.3096 (2)	0.0163 (5)	
C14	0.12487 (17)	0.5652 (3)	0.3880 (2)	0.0171 (5)	
H14	0.1197	0.5921	0.4577	0.020*	

### Atomic displacement parameters (Å^2^)

**Table:**

	*U*^11^	*U*^22^	*U*^33^	*U*^12^	*U*^13^	*U*^23^
Br1	0.02322 (15)	0.02735 (15)	0.01301 (13)	−0.00167 (11)	0.00141 (10)	−0.00143 (11)
Cl1	0.0119 (3)	0.0207 (3)	0.0259 (3)	−0.0026 (2)	0.0063 (2)	−0.0016 (3)
O1	0.0127 (9)	0.0334 (10)	0.0096 (8)	−0.0027 (8)	0.0032 (7)	−0.0009 (8)
O2	0.0184 (9)	0.0369 (11)	0.0111 (9)	0.0009 (8)	0.0027 (7)	−0.0044 (8)
O3	0.0149 (9)	0.0247 (10)	0.0149 (9)	−0.0035 (7)	0.0029 (7)	−0.0025 (8)
N1	0.0129 (10)	0.0201 (11)	0.0079 (10)	−0.0002 (8)	0.0000 (8)	−0.0028 (8)
N2	0.0148 (10)	0.0162 (10)	0.0113 (10)	0.0002 (8)	−0.0027 (8)	−0.0009 (8)
C1	0.0124 (12)	0.0151 (12)	0.0136 (12)	0.0003 (9)	0.0020 (9)	0.0018 (10)
C2	0.0162 (12)	0.0192 (12)	0.0122 (12)	−0.0016 (10)	0.0018 (10)	−0.0003 (10)
C3	0.0131 (12)	0.0181 (12)	0.0176 (12)	−0.0002 (10)	0.0000 (10)	0.0005 (11)
C4	0.0127 (12)	0.0188 (13)	0.0209 (13)	0.0007 (10)	0.0052 (10)	0.0017 (10)
C5	0.0167 (12)	0.0163 (12)	0.0150 (12)	0.0017 (10)	0.0054 (10)	0.0012 (10)
C6	0.0137 (11)	0.0127 (11)	0.0113 (11)	0.0010 (9)	0.0008 (9)	0.0021 (9)
C7	0.0168 (12)	0.0151 (11)	0.0116 (12)	0.0017 (10)	0.0018 (10)	0.0028 (10)
C8	0.0170 (12)	0.0144 (12)	0.0124 (11)	0.0013 (10)	0.0005 (10)	−0.0014 (10)
C9	0.0140 (12)	0.0147 (12)	0.0157 (12)	0.0005 (9)	0.0009 (10)	−0.0004 (10)
C10	0.0144 (12)	0.0133 (12)	0.0177 (12)	0.0022 (9)	0.0038 (10)	0.0040 (10)
C11	0.0185 (13)	0.0147 (11)	0.0132 (12)	0.0014 (10)	0.0011 (10)	0.0004 (10)
C12	0.0140 (12)	0.0152 (12)	0.0175 (13)	−0.0008 (10)	−0.0021 (10)	−0.0002 (10)
C13	0.0126 (12)	0.0132 (11)	0.0224 (13)	0.0007 (9)	0.0038 (10)	0.0010 (10)
C14	0.0188 (12)	0.0150 (12)	0.0166 (12)	0.0020 (10)	0.0037 (10)	0.0003 (10)

### Geometric parameters (Å, °)

**Table:**

Br1—C11	1.890 (3)	C3—H3	0.9500
Cl1—C13	1.749 (3)	C4—C5	1.387 (4)
O1—C1	1.361 (3)	C4—H4	0.9500
O1—H1O	0.833 (10)	C5—C6	1.404 (3)
O2—C7	1.229 (3)	C5—H5	0.9500
O3—C10	1.344 (3)	C6—C7	1.483 (3)
O3—H3O	0.836 (10)	C8—C9	1.459 (3)
N1—C7	1.353 (3)	C8—H8	0.9500
N1—N2	1.369 (3)	C9—C14	1.399 (4)
N1—H1N	0.872 (10)	C9—C10	1.412 (4)
N2—C8	1.279 (3)	C10—C11	1.393 (3)
C1—C2	1.395 (3)	C11—C12	1.382 (4)
C1—C6	1.399 (3)	C12—C13	1.383 (4)
C2—C3	1.382 (4)	C12—H12	0.9500
C2—H2	0.9500	C13—C14	1.379 (4)
C3—C4	1.384 (4)	C14—H14	0.9500
			
C1—O1—H1O	106 (2)	O2—C7—N1	121.6 (2)
C10—O3—H3O	107 (2)	O2—C7—C6	120.8 (2)
C7—N1—N2	118.7 (2)	N1—C7—C6	117.6 (2)
C7—N1—H1N	117.4 (18)	N2—C8—C9	120.0 (2)
N2—N1—H1N	123.5 (18)	N2—C8—H8	120.0
C8—N2—N1	117.0 (2)	C9—C8—H8	120.0
O1—C1—C2	121.0 (2)	C14—C9—C10	119.8 (2)
O1—C1—C6	119.0 (2)	C14—C9—C8	118.2 (2)
C2—C1—C6	120.0 (2)	C10—C9—C8	122.0 (2)
C3—C2—C1	120.2 (2)	O3—C10—C11	119.1 (2)
C3—C2—H2	119.9	O3—C10—C9	122.8 (2)
C1—C2—H2	119.9	C11—C10—C9	118.0 (2)
C2—C3—C4	120.7 (2)	C12—C11—C10	122.3 (2)
C2—C3—H3	119.6	C12—C11—Br1	118.63 (19)
C4—C3—H3	119.6	C10—C11—Br1	119.05 (19)
C3—C4—C5	119.4 (2)	C11—C12—C13	118.6 (2)
C3—C4—H4	120.3	C11—C12—H12	120.7
C5—C4—H4	120.3	C13—C12—H12	120.7
C4—C5—C6	121.0 (2)	C14—C13—C12	121.3 (2)
C4—C5—H5	119.5	C14—C13—Cl1	119.6 (2)
C6—C5—H5	119.5	C12—C13—Cl1	119.01 (19)
C1—C6—C5	118.7 (2)	C13—C14—C9	119.9 (2)
C1—C6—C7	125.3 (2)	C13—C14—H14	120.0
C5—C6—C7	116.0 (2)	C9—C14—H14	120.0
			
C7—N1—N2—C8	175.3 (2)	N2—C8—C9—C14	172.3 (2)
O1—C1—C2—C3	−179.7 (2)	N2—C8—C9—C10	−6.4 (4)
C6—C1—C2—C3	−0.5 (4)	C14—C9—C10—O3	−179.4 (2)
C1—C2—C3—C4	0.8 (4)	C8—C9—C10—O3	−0.7 (4)
C2—C3—C4—C5	−0.6 (4)	C14—C9—C10—C11	0.5 (4)
C3—C4—C5—C6	−0.1 (4)	C8—C9—C10—C11	179.2 (2)
O1—C1—C6—C5	179.1 (2)	O3—C10—C11—C12	179.1 (2)
C2—C1—C6—C5	−0.2 (4)	C9—C10—C11—C12	−0.9 (4)
O1—C1—C6—C7	−2.6 (4)	O3—C10—C11—Br1	−0.5 (3)
C2—C1—C6—C7	178.1 (2)	C9—C10—C11—Br1	179.55 (18)
C4—C5—C6—C1	0.4 (4)	C10—C11—C12—C13	0.6 (4)
C4—C5—C6—C7	−178.0 (2)	Br1—C11—C12—C13	−179.88 (18)
N2—N1—C7—O2	1.8 (4)	C11—C12—C13—C14	0.1 (4)
N2—N1—C7—C6	−178.3 (2)	C11—C12—C13—Cl1	179.70 (19)
C1—C6—C7—O2	−172.2 (2)	C12—C13—C14—C9	−0.5 (4)
C5—C6—C7—O2	6.1 (3)	Cl1—C13—C14—C9	179.98 (18)
C1—C6—C7—N1	7.9 (4)	C10—C9—C14—C13	0.1 (4)
C5—C6—C7—N1	−173.8 (2)	C8—C9—C14—C13	−178.6 (2)
N1—N2—C8—C9	−179.6 (2)		

### Hydrogen-bond geometry (Å, °)

**Table:**

*D*—H···*A*	*D*—H	H···*A*	*D*···*A*	*D*—H···*A*
O1—H1o···O2^i^	0.83 (1)	1.76 (1)	2.591 (2)	175 (3)
O3—H3o···N2	0.84 (1)	1.88 (2)	2.632 (3)	149 (3)
N1—H1n···O1	0.87 (1)	1.90 (2)	2.614 (3)	139 (2)

Symmetry codes: (i) *x*, −*y*+3/2, *z*+1/2.
